# Editorial: New Advances in Genetic Studies to Understand Yeast Adaptation to Extreme and Fermentative Environments

**DOI:** 10.3389/fgene.2021.663641

**Published:** 2021-03-11

**Authors:** Roberto Pérez-Torrado, Francisco A. Cubillos

**Affiliations:** ^1^Food Biotechnology Department, Instituto de Agroquímica y Tecnología de los Alimentos–Consejo Superior de Investigaciones Científicas, Valencia, Spain; ^2^Departamento de Biología, Facultad de Química y Biología, Universidad de Santiago de Chile, Santiago, Chile; ^3^Agencia Nacional de Investigación y Desarrollo (ANID)–Millennium Science Initiative Program–Millennium Institute for Integrative Biology (iBIO), Santiago, Chile

**Keywords:** yeast, adaptive evolution, extreme enviroment, fermentation, wine, beer

This Research Topic features recent advances in research on the mechanisms involved in yeast genetic adaptations. It highlights the tremendous importance of using and applying these advances to the enhancement and optimization of industrial fermentative processes. This *Frontiers eBook* includes studies related to the production of some of the most popular drinks, such as wine or beer, by conventional (i.e., *Saccharomyces* sp) and non-conventional yeast species (non-*Saccharomyces* sp). In particular, it includes recent work on the study of yeast physiological adaptations to extreme and fermentative environments aided by the increasing power of new high-throughput phenotyping and sequencing technologies, the latter including genome re-sequencing projects and RNA-seq studies. Yeast adaptations to those environments increases industrial yeasts' diversity and comprises several genetic mechanisms, including horizontal gene transfer (HGT), chromosomal rearrangements, hybridizations, and genome duplications. A literature review on these mechanisms is included in this Research Topic (Giannakou et al.).

An area of significant interest is the physiological study and optimization of yeast to brewing environments. The collection includes a study that highlights the potential of non-conventional yeast, such as *Lachancea fermentati* strains isolated from Kombucha, to produce low alcohol beer (Bellut et al.). Bellut et al. demonstrated the potential of *L. fermentati* to substantially decrease ethanol concentration in beer due to the metabolic deviation toward the production of lactic acid. This work attracted significant attention because of its impact on the generation of low-alcohol drinks, a topic that is currently of research interest in the industry.

In a study focused on brewing yeast optimization, de Vries et al. describe an elegant new methodology to generate strain diversity based on engineering chromosome copy number variation (CCNV) through centromere-silencing. The authors noticed that variable CCNV in industrial *Saccharomyces* generates phenotypic diversity, allowed them to select for ethanol tolerant mutants. Another study in this collection, examining yeast tolerance to high ethanol concentrations in high gravity fermentations, suggests that this tolerance is dependent on the unfolded protein response and proteostasis (Telini et al.), a finding that is in agreement with previous reports (Miyagawa et al., 2014; Navarro-Tapia et al., 2017).

Another topic covered in this *Frontiers eBook* is the identification of genetic and molecular evidence of adaptation and optimization of *Saccharomyces cerevisiae* yeast strains to wine environments. A pivotal study by Devia et al. demonstrates the role of new genes acquired through HGT in wine fermentation. Here, the authors carried out a systematic analysis of the promoter activity and protein levels of 30 genes located within three different horizontally acquired regions found across various wine strains. The authors highlight ORF A9, an ortholog of a thiamine (vitamin B1) transporter, which appears to impact biomass and fermentation capacity traits. These findings are complemented in this collection with a literature review on the adaptive advantages provided by an oligopeptide transporter family acquired in *S. cerevisiae* wine strains by horizontal gene transfer from *Torulaspora microellipsoides* (Becerra-Rodriguez et al.).

Chromosomal rearrangements around *SSU1* promoters are known to provide higher sulfite tolerance to enological *S. cerevisiae* strains under wine environments. In this context, Marullo et al. applied a novel multiplex PCR method (*SSU1* checkup) to analyze nearly 600 wine strains from vineyards and cellars, observing an enrichment of rearranged *SSU1* promoters in commercial strains.

Another hallmark of wine yeasts is efficient nitrogen assimilation. Molinet et al. studied the different *S. cerevisiae* allelic variants of *GTR1*, a GTPase that participates in the EGO complex responsible for *TORC1* activation in response to amino acids availability. The authors conducted a series of elegant experiments, including allelic swapping, and demonstrated that polymorphisms in the coding region of *GTR1* in a specific subpopulation were relevant for TORC1 activity, impacting nitrogen consumption under wine fermentation conditions. In another study on vineyard-associated environments, conducted by Cheng et al., the authors reported a predominance of *S. cerevisiae* and some *S. uvarum* strains in spontaneous Pinot Noir fermentations. The authors identified a specific anthocyanin and flavonoid profile in fermented wines representative of this viticultural area. In another study included in the collection, the molecular underpinning of the velum stage, which is particular to sherry-wines, was also investigated, via RNA-seq (Mardanov et al.). The authors found a complex response during biofilm formation in response to this stressful environment, including strong upregulation of the flocculation gene *FLO11*. Altogether, these studies demonstrate how genetic hallmarks in different yeast strains are responsible for their improved fermentative capacity.

This Research Topic also includes studies on non-conventional yeasts and how HGT can mediate the reestablishment of alcoholic fermentation. The study by Gonçalves et al. on the evolutionary history of the *Wickerhamiella* and *Starmerella* genera (W/S clade) demonstrates that these fructophilic species have a preference to produce mannitol to compensate for a probable ancient loss of capacity to perform alcoholic fermentation.

Finally, the collection includes a review on the cellular responses induced by Msn2-mediated proline incorporation, which represents an important yeast adaptation to many suboptimal environmental conditions (Mat Nanyan and Takagi).

This Research Topic highlights recent key findings on yeast adaptations to extreme and industrial environments. Besides the crucial basic knowledge reported and discussed here, these discoveries can help various industrial processes, such as wine or beer production, by allowing the enhancement and optimization of the yeasts involved in popular food products.

## Author Contributions

RP-T and FC fulfill the standard requirements for authorship and read and approved the final manuscript. Both authors contributed to the article and approved the submitted version.

## Conflict of Interest

The authors declare that the research was conducted in the absence of any commercial or financial relationships that could be construed as a potential conflict of interest.
